# Comparative Study of Ochratoxin A Exposure through the Intake of Cereal Products in Two Climatic Moroccan Regions

**DOI:** 10.3390/toxins15070452

**Published:** 2023-07-09

**Authors:** Ahmed Tabarani, Abdellah Zinedine, João Miguel Rocha, Moez Sanaa, El Hassane Abdennebi

**Affiliations:** 1 Department of Biological and Pharmaceutical Sciences, Hassan II Institute of Agronomy and Veterinary Medicine (IAV), Rabat P.O. Box 6202, Morocco; ahmed.tabarani@gmail.com (A.T.); pr.abdennebi@yahoo.fr (E.H.A.); 2 BIOMARE Laboratory, Faculty of Sciences P.O. Box 20, Chouaib Doukkali University, El Jadida 24000, Morocco; zinedineab@yahoo.fr; 3 CBQF—Centro de Biotecnologia e Química Fina—Laboratório Associado, Universidade Católica Portuguesa, Escola Superior de Biotecnologia, Rua Diogo Botelho 1327, 4169-005 Porto, Portugal; 4 LEPABE—Laboratory for Process Engineering, Environment, Biotechnology and Energy, Faculty of Engineering, University of Porto, 4200-465 Porto, Portugal; 5 ALiCE—Associate Laboratory in Chemical Engineering, Faculty of Engineering, University of Porto, 4200-465 Porto, Portugal; 6 Risk Assessment Department, French Agency for Food, Environmental and Occupational Health & Safety (ANSES), 94701 Maisons-Alfort, France; moez.sanaa@gmail.com

**Keywords:** ochratoxin A, cereal derivatives, climate, exposure, risk assessment, regulations, Morocco

## Abstract

The present study aims to compare ochratoxin A (OTA) exposure through the intake of three cereal derivative products (bread, pasta and semolina) in two different Moroccan climatic regions (littoral and continental). OTA weekly intakes from cereal products were calculated using a deterministic approach for each region. Results showed a statistically significant difference (*p* < 0.05) of OTA exposure between the two regions. Indeed, the median OTA exposure was estimated at 48.97 ng/kg b.w./week in the littoral region, while it was estimated at 6.36 ng/kg b.w./week in the continental region. The probabilistic approach showed that, due to uncertainties, the 95th percentile of weekly OTA exposure associated with the three cereal products ranged from 66.18 to 137.79 (95% CI) with a median of 97.44 ng/kg body weight (b.w.)/week. Compared to the threshold of 100 ng/kg b.w./week, 95% of the cumulative distributions predicted an exceedance frequency between 0.42 and 17.30% (95% CI), with an exceedance frequency median of 4.43%. Results showed that cereal derivatives constitute an important vector of OTA exposure and cause a significant exceedance of toxicological reference value among large consumers in the littoral region, which suggests the urgency of reconsidering the maximum regulatory limit (MRL) set for OTA (3 µg/kg) in cereal derivatives by Moroccan authorities.

## 1. Introduction

Mycotoxins are chemical secondary metabolites of different fungi and are considered as natural contaminants of cereals, so their presence is often inevitable. The most common mycotoxins of concern to humans and livestock include aflatoxins, citrinin, ochratoxins, fumonisins, patulin, zearalenone, nivalenol, deoxynivalenol, fumonisins, and ergot alkaloids [1], and aflatoxin B1 (AFB1) is confirmed as carcinogenic to humans and animals [2].

Ochratoxins are among the worrying mycotoxins and are natural metabolites of toxigenic fungal species of the genus *Aspergillus* and *Penicillium*, mainly produced by *Penicillium verrucossum* in temperate climates and by *Aspergillus ochraceus* in warm regions [3]. Ochratoxin A (OTA) is the most toxic of the ochratoxins [4]. OTA was isolated in 1965 and chemically characterized [5]. Its chemical formula contains a molecule of 3-methyl-5-chloro-8 hydroxy-3,4 dihydroisocoumarin linked by a peptide bond, at the level of its C7 carboxyl group, to the amine group of L-β-phenylalanine [6]. Recent reports have stated that black *Aspergilli* species (*A. niger* aggregate and *A. carbonarius)*, are sources of OTA in food products, especially cereals [7] and other dried foods, such as dried palm dates [8].

Toxicological studies on laboratory animals have shown that this toxin may have several potential effects, such as nephrotoxicity, genotoxicity, immunosuppression, teratogenicity, neurotoxicity and carcinogenicity [9]. Recent reports revealed a proven carcinogenic effect of OTA on the kidneys, liver and intestine in different laboratory animals [10]. Although OTA was associated for a long time to Balkan endemic nephropathy (BEN) disease [11], recent evidence indicates that this pathology is due to aristolochic acid as the causative agent [12].

The genotoxicity of OTA is still debated and there is not enough strong evidence to establish its genotoxicity; some studies suggest that OTA carcinogenicity may be due to mechanisms other than direct DNA damage, such as oxidative stress or epigenetics changes [13]. It has been classified by the International Agency for Research on Cancer (IARC) as a possible human carcinogenic in group 2B [14]. A provisional tolerable weekly intake (PTWI) was set by the Joint FAO/WHO Expert Committee on Food Additives (JECFA) for humans at 100 ng OTA/kg bodyweight (b.w.) per week (i.e., 14 ng/kg b.w./d), based on nephrotoxic effects observed in pigs in a 90-day sub-chronic toxicities study [15].

The European food safety authority (EFSA) derived a PTWI of 120 ng OTA kg body weight per week (i.e., 17 ng OTA/kg b.w./day) by adopting an approach based on the existence of a threshold of toxicity in the absence of conclusive evidence that OTA binds to DNA. The PTWI was derived based on the lowest-observed-adverse-effect level (LOAEL) of 8 µg/kg b.w./day for early markers of renal toxicity in pigs (the most sensitive animal species), and applying a composite uncertainty factor of 450 for uncertainties in the extrapolation of experimental data from animals to humans, as well as for intra-species variability [9]. More recently, based on new studies, the EFSA adopted a benchmark dose (BMDL10) of 4.73 µg/kg b.w./day calculated from the renal lesions observed in pigs for the characterization of non-neoplastic effects. For characterization of neoplastic effects, a BMDL10 of 14.5 µg/kg b.w./day was calculated from renal tumors observed in rats [16].

Humans are exposed to OTA through consumption of food products contaminated either during primary production or during storage [17]. OTA can be inhaled in dust particles or spores from contaminated grains and can present a risk for workers in the agricultural and agri-food industry. Although OTA can be absorbed through the skin, this exposure pathway is less common. OTA has been found mainly in cereals (wheat, corn, rice, rye and oats), coffee, cocoa, beans, peas, peanuts and dried fruits, and offal and meat from animals fed with contaminated feed [18]. Due to its thermal stability, OTA removal or reduction from contaminated processed cereals is very difficult. Consequently, humans are frequently exposed to OTA through cereal products intake [19]. Recently, several reports have been published on OTA exposure through food and cereal intake, especially from China [20], Ghana [7], Chile [21], Vietnam [22], Lebanon [23], and Tunisia [24] etc.

In Morocco, several studies have reported the occurrence of OTA in various food products including cereals and their derivatives [25,26,27,28,29,30,31,32]. Morocco’s food consumption model is still largely dominated by cereals, mainly soft wheat. The latter is consumed at every meal, as a side to the basic tajine dish, a vegetable and/or legume-based stew, with or without meat [33].

New regulations have been adopted by Moroccan authorities to set maximum regulatory limits (MRL) of certain mycotoxins in foodstuffs due to their associated health and economic negative impacts. These regulations have recently been repealed and modified by the annex of the Joint Decree n°2410-22 of 14 September 2022 [34]. Thus, Moroccan regulations have set the MRL for OTA at 5 and 3 µg/kg in raw cereals and cereal derivatives, respectively, which are similar to those previously adopted by the European Union in 2006 and repealed in 2023 [35].

More recently, the European Union has adopted new regulations (EC No 1370/2022) for OTA in foodstuffs for which no maximum level has been established yet, and which contribute to OTA human exposure. Thus, the MRL of OTA in bakery wares, cereal snacks and breakfast cereal has been lowered by European countries to 2 µg/kg, since these products are considered as major vectors of OTA human exposure [36].

On average, a Moroccan consumes 185 kg of cereals per year [37], compared to a world average of 152 kg [38]. A recent study of our research group showed that bread and cereal derivatives can constitute, under certain scenarios, an important vector of the human exposure to OTA [39]. However, this study focused on national average OTA contributions, and the regional contributions were not considered. As an extension, the regional factor was included in the present study since Morocco, a Mediterranean and North African country, is known for its climate with high-annual variations in temperature and recorded rainfall, and has several different climatic conditions that may influence mycotoxin levels in cereals and derivatives, resulting in an uneven distribution of risk between regions.

Fungal infection and colonization, as well as mycotoxin production, are affected by both biotic (grain maturity, pest damage, fungal species) and abiotic factors, such as temperature, water stress, relative humidity and water activity. These factors are responsible for infection and colonization of cereals by mycotoxigenic fungi, and further contamination with mycotoxins. It has been assumed that fungal growth and mycotoxin synthesis depend on the environmental and meteorological conditions which vary from year to year and region to region. This climatic variability can lead to the infection of cereal crops by toxigenic fungi, which then produce mycotoxins and influence the quality of the harvest [40].

Thus, the purpose of this study is to assess Moroccan adults’ exposure to OTA from the consumption of three cereal products (bread, pasta and semolina) collected in two different climatic areas (continental and littoral) in the country, and to check if these products, by their own, might lead to exceedances of the toxicological reference dose of JECFA (PTWI, 100 ng/Kg b.w./week) in the two Moroccan regions, where the climate differs considerably.

## 2. Results

### 2.1. Analytical Results

Results of OTA levels in analyzed samples of cereal products are provided in Table 1. As shown, the maximum OTA levels were registered in samples of semolina (14.13 µg/kg) and bread (7.2 µg/kg) collected from Tétouan (littoral) and Marrakech (continental), respectively.

In the littoral region, the mean contamination OTA level of bread was higher (1.32 ± 0.53 µg/kg) compared to semolina and pasta. While in the continental region, the mean OTA level in bread remains much lower (0.05 ± 0.03 µg/kg). In this region, semolina recorded the highest mean level of OTA contamination (0.61 ± 0.80 µg/kg). Overall, and apart from the type of cereal derivative, the Littoral region recorded an average OTA contamination of 0.82 ± 0.06 µg/kg, which is high compared to the continental one (0.36 ± 0.01 µg/kg). In this region, the percentage of contaminated samples remains low (16.1%), compared to 45.3% registered in the littoral.

Regarding the regulatory limits set for OTA in cereal derivatives, there were eight bread samples (7%) from the littoral area and two semolina samples (1.7%) from the continental area that exceeded the MRL of 3 µg/kg set by European legislation and Moroccan authorities [34].

### 2.2. Deterministic Approach

The EWI (Exposure Weekly Intake) was deterministically calculated for different percentiles of consumption as 25th percentile (P25), 50th percentile (P50), 75th percentile (P75) and 95th percentile (P95) for the two regions based on the obtained data (Table 2). The mean contamination was used in all exposure calculations as it represents a good estimate of long-term contamination, and because the objective is to assess chronic OTA exposure.

The estimated median exposure was 48.97 ng/kg b.w./week in the littoral region, which represents 48.97% of the PTWI. In the continental region, the observed median of exposure was estimated at 6.36 ng/kg b.w./week, equivalent to 6.36% of the PTWI. In the littoral region, cereal derivatives contribute significantly to OTA exposure, compared to the continental region, where this contribution remains much lower (more than seven times less).

At the highest percentile (P95), it is noted that the PTWI is exceeded among large consumers of cereal derivatives in the littoral region (114.3%). Bread alone contributes for 103.7%. This exceedance of the PTWI is observed even for low levels of consumption of semolina and pasta (P25). In contrast, in the continental region, the EWI remains, in the vast majority of cases, significantly lower than the EWI in the littoral region (*p* < 0.05).

### 2.3. Probabilistic Approach

The probabilistic calculation was carried out for the littoral region, where the PTWI was exceeded at the highest percentile (114.3 ng/kg b.w./week).

**Figure 1:**
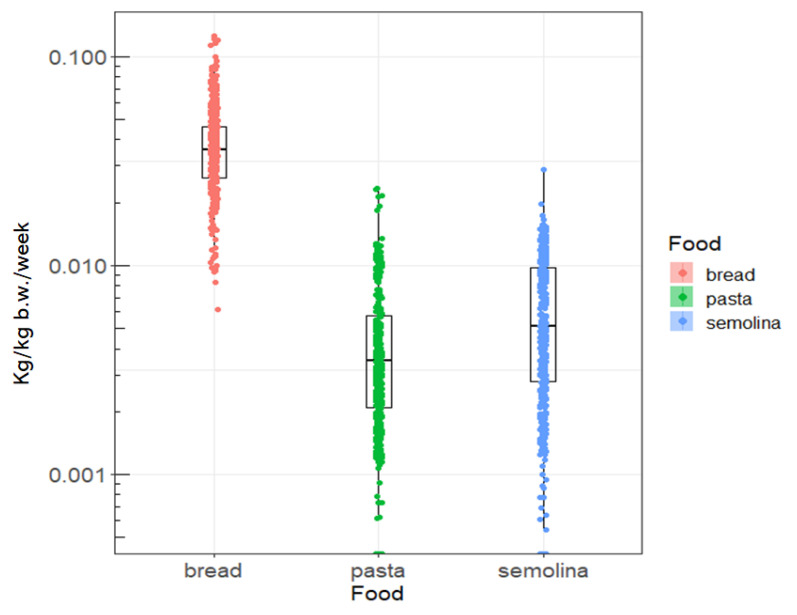
Distribution of weekly consumption (Kg/kg b.w./week) of cereals derivatives observed in the littoral region.

**Figure 2:**
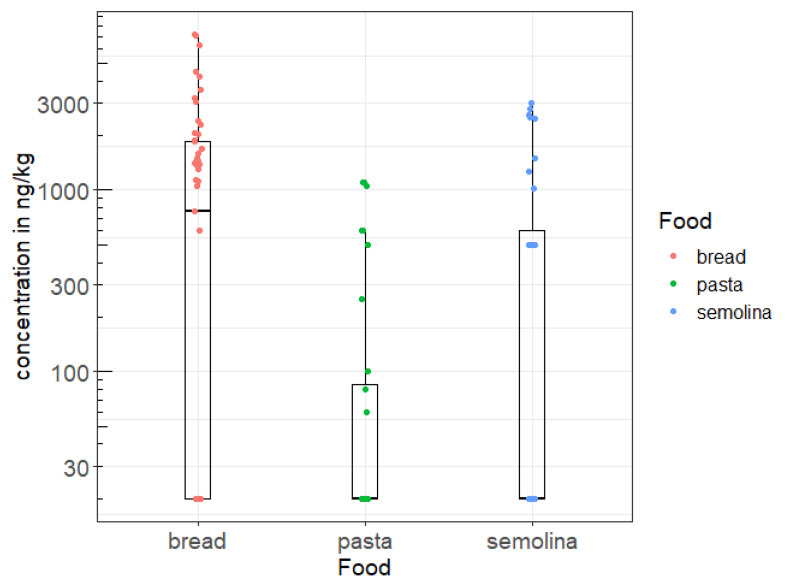
Distribution of OTA concentrations (ng/kg) observed in the littoral region.

Figure 1 and Figure 2 show the distribution of weekly consumption of the three foods, as well as the observed OTA concentrations. Thus, it seems that the mean consumption of cereal derivatives in the littoral region is higher compared to the national average which is 0.03 kg/kg b.w./week. This is probably due to the fact that dietary habits and the standard of living in this region are where these products still constitute the basic ration. In addition, bread seems to be the most OTA-contaminated cereal derivative, compared to the other cereal products investigated in this study, namely semolina and pasta (Figure 2). This level of bread contamination observed in the littoral region is also higher than the national average contamination level (0.28 µg/kg) previously reported by Tabarani et al. [39].

**Figure 3:**
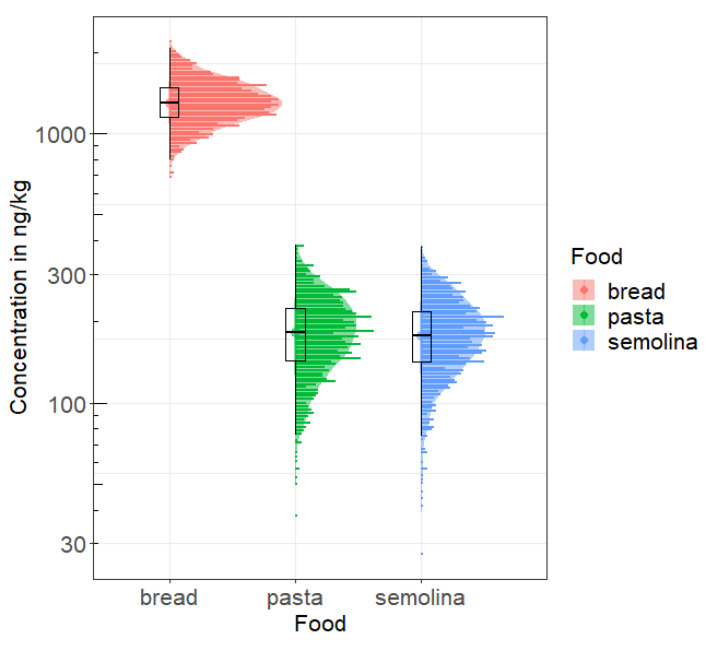
Distribution of average OTA concentrations (ng/kg) for the three cereal derivatives obtained with the 1000 draws with presentation of the results observed in in the littoral region.

Figure 3 shows the distribution of the average OTA concentrations for the three foods obtained with the 1000 draws with replacement, and illustrates both variability and uncertainty. 

**Figure 4:**
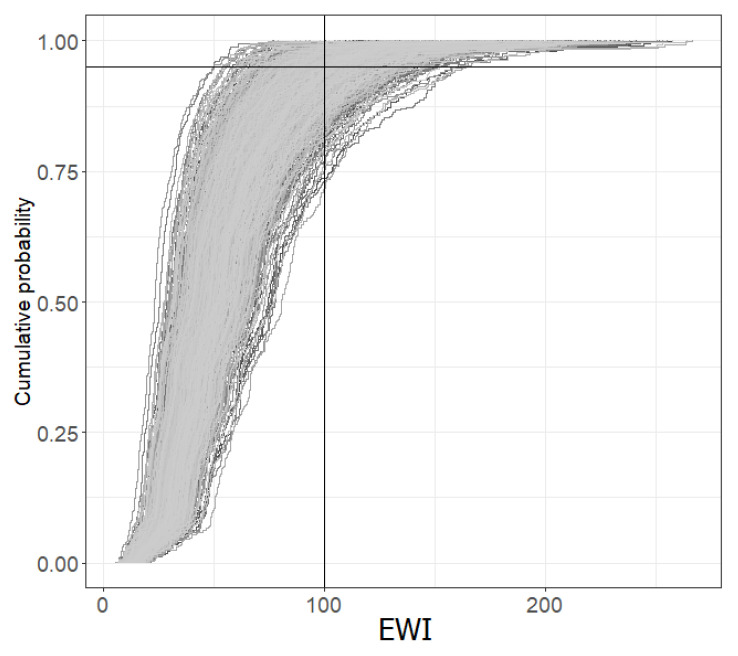
Cumulative distribution of EWI—Probabilistic approach.

Figure 4 shows the cumulative distribution of OTA exposures associated with the consumption of the three foods. Indeed, in this figure, one can have the superposition of 1000 cumulative distributions, each illustrating the variability linked to the differences in the consumption of the three foods between individuals, whereas the differences between these thousand distributions illustrate the uncertainty associated with the sampling of individuals and analyzed food. The percentiles of the EWI were derived from the cumulative distributions shown in this figure.

Table 3 provides the P50, P75 and P95 of the probabilistic EWI with their confidence interval (representing the uncertainty). The 95th percentile of weekly OTA exposure associated with the three foods (ng/kg body weight) is estimated to be between 66.18 and 137.79 (95% CI), with a median of 97.44.

Compared to the PTWI of 100 ng/kg b.w./week, the frequency of exceedance is estimated between 0.42 and 17.30% (95% CI), with a median of exceedance frequency of 4.43%. The exceedance of the PTWI is essentially due to the consumption of bread. Indeed, the frequency of exceedance changes only very little when the exposures linked to the other two foods are eliminated.

## 3. Discussion

Cereal derivatives are the most important source of food for the Moroccan population and around the world, i.e., it represents a staple food. This population continues to grow with an increasing demand for these commodities. In the country, harvested or imported cereals are generally stored in specific conditions to guarantee a continuous supply throughout the year. During storage, economic losses can become very significant due to the reduction in grain quality. Grain spoilage caused by fungal contamination results in grain loss that can occur during storage, pre-harvest and post-harvest conditions, which are controlled by environmental factors such as water activity (a_w_), temperature and rainfall. The influence of climatic conditions and OTA production has been reported [41]. Furthermore, it has been shown that elevated CO_2_ concentration in the Mediterranean climate may result in an increased risk of OTA contamination [42]. Conversely, Cheng and Van der Fels-Klerx reported that the risk of OTA production in cereals may increase as a result of inadequate storage and transport conditions across changing climate zones [43].

The higher consumption of bread compared to pasta and semolina leads to increased exposure of the inhabitants of the littoral region to high levels of OTA. This exposure appears to be higher than that observed in other countries from similar products. Indeed, this exposure was observed at 0.30 ng/kg b.w./day in Portugal [44], at 0.36 ng/kg b.w./day in Germany [45], at 1.6 ng/kg b.w./day in Spain [46], at 1.63 ng/kg b.w./day in Canada [47] and at 5.0–24.9 ng/kg b.w./day in Tunisia, indicating a high degree of OTA exposure in the Tunisian population [24].

In Lebanon, where the climate is similar to Morocco, especially in the costal Mediterranean zones, the total OTA exposure in large consumers (95th percentile) was estimated from 47 food products at 13.6 ng/kg b.w./day [48], a value very close to the PTWI of JECFA (i.e., 14 ng/kg b.w./day). The percentage contribution of cereal derivatives to this exposure was 35.2%, or approximately 33 ng/kg b.w./week, which remains lower than that observed in the Moroccan coastal region. This could be attributed to the consumption level of cereal derivatives, which remains different in the two countries. Furthermore, the Joint FAO/WHO Committee (JECFA) has established in 2008, an exposure of 1.14–2.43 ng/kg b.w./day from the same products based on European data. Indeed, EFSA estimated a maximum OTA exposure from several foods that ranges from 2.53 to 17.79 ng/kg b.w./day. The largest contributors to this dietary exposure to OTA were canned meat, cheese, and cereals and cereal-based products [16].

The significantly high average OTA intakes in the littoral region seems to be partly linked to the OTA contamination level of bread, which remains the most consumed cereal derivative. The climate of this region seems to offer favorable conditions for a significant production of this mycotoxin. High-OTA levels were previously reported in bread collected from the region of Casablanca (coastal area), located on the Atlantic Ocean (13.6 µg/kg) by Zinedine et al. [29].

Bread seems to be the most important vector of exposure to OTA in the littoral region, where exceeding the PTWI was observed. This important contribution of bread to the exposure was also reported in the second total diet study conducted by ANSES [49], which showed that bread and dry bread products appear as the main contributor to OTA exposure (20 and 80%, respectively) in France.

For the estimation of exposure using the deterministic approach, it was assumed that the consumption of the three food products were independent, which is not the case. It is indeed unlikely that an individual will consume the maximum quantity of all the foodstuffs considered. This simplification leads to an overestimation of OTA exposure. The probabilistic approach considers the issue of overestimation, as it estimates the exposure on an individual-by-individual basis. In addition, the used probabilistic approach allowed the consideration of both variability and uncertainty. It should be clarified that the probabilistic approach of risk assessment remains a rigorous technique and its use provides the means to improve risk management decisions. However, given the increased analytical demands associated with the use of this technique, probabilistic risk assessment should only be used when the results will influence decision-making [50].

The estimated percentiles of exposure are lower than the one estimated with the deterministic approach. However, the probabilistic approach is based only on 24 h recall of food consumption and ignores the intra-individual variability that might still lead to a certain overestimation of the EWI.

Because bread is a daily staple food, it is expected that the intra-individual variability will not be very large. Consideration of intra-individual variability should not substantially reduce the estimated exposures. In any case, the results are rather robust with respect to the relevance of bread as a source of OTA exposure in the littoral region.

Overall, it is strongly recommended that measures be taken to prevent contamination of bread in this region through good agricultural practices, cereal storage practices, and hygiene and bread-making practices.

With a view to protect Moroccan consumers, we recommend that regulatory authorities should revise the national mycotoxin regulations to lower the MRL of OTA from 3 to 1 µg/kg in bakery products, in order to protect large consumers, especially in littoral regions where OTA exposure could be high. Consumption of bakery products, especially bread, is very important by Moroccan households. This downward revision should reduce the contribution of these products to human OTA exposure by 32% in large consumers of cereal derivatives in the country. The new OTA regulation EC No 1370/2022, adopted by European countries by lowering the MRL from 3 to 2 µg/kg in bakery products, was based specifically on new contamination data. The EFSA considered that it was not necessary to establish a guideline value for health protection for OTA, and that the tolerable weekly intake of 120 ng/kg b.w./week, as established in 2006, was no longer valid. It further concluded that the calculated margin of exposure (MOE) for the carcinogenic effects of OTA indicate that it could pose a health concern for certain groups of consumers [16].

Regarding the methodology for risk characterization associated with OTA exposure, and given that the genotoxic status of OTA is very controversial, it would be interesting to examine this aspect under the assumption that OTA is genotoxic and therefore acts without a toxicity threshold.

Indeed, EFSA [51], Barlow et al. [52] and Kuiper-Goodman et al. [47] claim that OTA should be regulated as a genotoxic substance. This remains valid in the case of substances which are carcinogenic but whose carcinogenic mode of action has not been identified. This is actually a default position based on a lack of other information.

We will thus propose that the present work should be analyzed according to an approach adapted to this new hypothesis, such as the MOE, which is applied for risk characterization associated with substances which act without a toxicity threshold. It would be interesting to check if the risk for the consumers remains unchanged in the two approaches or if an underestimation has been produced.

Finally, according to recent investigations, the presence of other components, mainly, the co-presence of mycotoxins in cereal derivatives, could affect the final bioavailability of mycotoxins, including OTA [53]. it would be interesting to evaluate the bioaccessibility and the bioavailability of OTA in order to estimate, by toxicokinetic data modeling (in vitro and in vivo models), the internal contributions of OTA that pass into the bloodstream and induce the harmful effects associated with this mycotoxin.

## 4. Conclusions

In conclusion, this study shows that cereal derivatives constitute an important vector of exposure to ochratoxin A, particularly in coastal regions with humid and temperate climates. The risk of developing harmful effects of OTA would be higher in these regions compared to the Moroccan arid regions. Cereal products alone are capable of causing a significant overrun of the tolerance reference value among heavy consumers of these derivatives in coastal regions. In this regard, we underline the importance of reconsidering the maximum limits of OTA in cereal derivatives set by national regulations. The methodology used could be further refined to consider Moroccan dietary habits.

## 5. Materials and Methods

Estimating exposure to food contaminants is an activity that remains complex, and no single approach is suitable for all circumstances. The method chosen depends on the information available, the population group concerned, the evaluation of the acute or chronic effects of the contaminant and the intended use of the result [54].

Several methods are reported in the literature on the estimation of consumer exposure to food contaminants. The application of these methods requires: (1) Availability of contamination data reporting the levels of chemicals in the food vectors; (2) Availability of the official consumption data of foods by age groups or population groups; and (3) the use of an estimation method that combines contamination data with consumption data. The data combination can be carried out according to different approaches: (a) a deterministic approach; (b) a simple distribution; or (c) a probabilistic approach.

The present study included three Moroccan cereal products, namely: bread, pasta and semolina. These three products were taken from the local market in the two regions of Tétouan (littoral) and Marrakech (continental), where the climate considerably differs. The littoral region is a coastal and Mediterranean area located in North Morocco with a humid and temperate climate (annual mean temperature: 17.2 °C; precipitation: 585 mm; and annual humidity level: 54), and the continental region is located in the center of Morocco with a dry and arid climate (annual mean temperature: 18.5 °C, precipitations: 288 mm; and annual humidity level: 75).

The EWI of OTA from the three considered food products were calculated for consumers, according to the following Equation (1) [55,56]:

(1) $$EWI=\sum_{i=1}^{3}\frac{C_{i}\times Qte_{i}}{B.W.}$$

where:

₋ *EWI*: the estimated weekly intake associated with the consumption of the three foods (i: 1: bread, 2: pasta, 3: semolina) in ng/kg of body weight/week (ng/kg b.w./week);
₋ *C_i_*: the observed mean of OTA concentration for each of the foods *i* in µg/Kg;
₋ *Qte_i_*: a percentile or average of the average quantity of food consumed per week per individual in g; and
₋ *B.W.*: the mean body weight of an individual in kg (70 kg).

The EWI was estimated according to the assumptions that OTA contamination level of the three cereal derivatives is relatively stable over time; individuals do not change their eating habits much and, therefore, the intra-individual variability in the consumption of cereal derivatives is relatively low; and, finally, consumption data for bread, pasta and semolina are independent.

The calculation of the EWI was determined among high consumers using the 95th percentiles of the consumption of the three considered foods. If the PTWI was exceeded, a probabilistic calculation was conducted by integrating both the variability between the consumption of individuals and the uncertainties linked to the sampling of foods and interviewed individuals on their consumption of the three foods (bread, semolina and pasta). The calculation followed the following steps:

₋ A total of 1000 draws are made with replacement (replications) from all of the results obtained on different foods. At each of the replications, the mean of the concentrations is calculated. Thus, 1000 averages for each of the three elements are obtained;
₋ The consumption table, which is made up of 474 rows (individuals) and 4 columns, and considers the weight of the individuals, means that the weekly consumption of bread, pasta and semolina was replicated 1000 times, each time carrying out 474 draws with replacement of each row of the table. A total table of 474,000 rows is obtained;
₋ For each of the lines, the weekly exposure to OTA is calculated by summing the products of the weekly quantity of the food by its average concentration of OTA, then dividing this sum by the weight. The average of the OTA concentrations is chosen from among the 1000 values already calculated, with respect to the replication number (1 to 1000).

To characterize the risk of OTA, we compared the EWI to the PTWI set by JECFA at 100 ng/kg b.w./week (or 14 ng/kg b.w./day) [57]. JECFA derived the PTWI based on a nephrotoxicity study in pigs [11], the most sensitive species, using the lowest dose tested (8 µg OTA/kg b.w./day) and applying a safety factor of 500. At this date, there is no information available that JECFA has reviewed its opinion on the mechanisms of genotoxicity of OTA, unlike EFSA. According to JECFA, there is no conclusive evidence that OTA binds to DNA and there is suggestive evidence regarding the role of oxidative processes, such as lipid peroxidation. Several reports have reached a similar conclusion [16,58,59].

### 5.1. Contamination Data

A total of two hundred twenty-six (*n* = 226) samples of cereals derivatives, including bread (*n* = 90), pasta (*n* = 59) and semolina (*n* = 77), were randomly collected in different conditions from the continental (*n* = 118) and littoral (*n* = 108) regions. All samples were ground and thoroughly mixed to <1 mm, divided into subsamples of 100 g and stored at 4 °C until OTA analysis. OTA was extracted from cereal derivatives samples and analyzed by a Shimadzu (Kyoto, Japan) system LC 1445 coupled with fluorescence detection (LC-FD), according to an internal validated method previously reported by Tabarani et al. [39]. The detection (LOD) and quantification (LOQ) limits that were reached by the employed method were 0.017 and 0.05 µg/kg, respectively. The contamination levels of the three cereal derivatives by OTA were estimated by applying the recommendations of GEMS/FOOD on left-censored data [60]. This estimation was made under the two usual assumptions:

₋ A low hypothesis (Lower Bound: LB): the undetected concentrations (ND) are replaced by 0 and the unquantified ones (NQ) have been substituted by the detection limit (LOD).
₋ A high hypothesis (Upper Bound: UB): the ND are replaced by the detection limit (LOD) and the NQs by the qualification limit (LOQ).

### 5.2. Consumption Data

The consumption data comes from a survey conducted using the 24 h recall method. A single 24 h recall was carried out on 474 subjects surveyed at the entrance of a supermarket during the weekend, located in the center-region. A consumer survey questionnaire has been developed for this purpose. Respondents were asked to recall the portions consumed of the three cereal derivatives studied during the day before the survey. Photographs illustrating different portions that can be consumed were used as a support to estimate consumption quantities. The frequencies of consumption of these products were also noted, which made it possible to estimate the quantities consumed during a whole week. The consumers were asked to provide an estimate of their body weight in kg.

## Figures and Tables

**Table 1:** OTA levels (µg/Kg) in analyzed cereal derivatives samples.

Sampling Area	Cereal Product	Sample Size	Minimum	Mean UB ^1^	Maximum
Littoral					
	Bread	44	0.02	1.32 ± 0.53	7.2
	Semolina	32	0.02	0.64 ± 0.34	3
	Pasta	32	0.02	0.18 ± 0.12	1.1
				0.82 ± 0.06 ^2,3^	
Continental					
	Bread	46	0.02	0.05 ± 0.03	0.5
	Semolina	45	0.02	0.61 ± 0.80	14.13
	Pasta	27	0.02	0.56 ± 0.26	2.1
				0.36 ± 0.01 ^2,3^	

^1^ Mean contamination level obtained under a high hypothesis (Upper Bound: UB) was retained because no statistical difference was observed between mean UB and mean LB (Lower Bound). ^2^ Average contamination of the three cereal derivatives. ^3^ Significant difference (*p*-value = 0.0062) between the mean contamination levels (littoral versus continental).

**Table 2:** Exposure (in ng/kg b.w./week) for different percentiles of consumption.

Sampling Area	Cereal Derivative	P25	P50	Mean	P75	P95
	Bread	37.71	45.26	52.33	56.57	103.7
Littoral	Semolina	1.40	3.20	3.95	6.40	8.69
	Pasta	0.26	0.51	0.70	0.90	1.93
	Total EWI	39.37	48.97	56.98	63.87	114.3
	Bread	1.43	1.71	1.98	2.14	3.93
Continental	Semolina	1.33	3.05	3.76	6.10	8.28
	Pasta	0.80	1.60	2.18	2.80	6.00
	Total EWI	3.56	6.36	7.92	11.04	18.21
*p*-value ^1^		0.0013	0.009	0.019	0.017	0.027

^1^: *p*-value between littoral and continental EWI for the different percentiles (P25, P50, P75 and P95) and the mean.

**Table 3:** Exposure (in ng/kg b.w./week) according to the probabilistic approach for different percentiles of consumption.

Exposure	CI(95%)	Median
Mean	[35.45–72.48]	51.88
P50	[32.60–67.38]	48.22
P75	[42.58–86.38]	61.81
P95	[66.18–137.79]	97.44

## Data Availability

Data presented in this study are available in the article.
